# Flu Vaccine Production Gets a Shot in the Arm

**DOI:** 10.1289/ehp.114-a108

**Published:** 2006-02

**Authors:** Ernie Hood

The world is holding its collective breath. Governments and health organizations are feverishly preparing, stockpiling drugs and vaccines, and formulating contingency plans. Headlines are coughing out dire predictions of up to 100 million deaths worldwide and devastating economic consequences. Experts are chillingly warning that it’s not a matter of if, but when—when a pandemic of avian influenza will strike the human population.

Although the H5N1 avian flu is already a serious problem in Asia, it will not become a major threat to human health worldwide until and unless the virus mutates into a strain that is both highly virulent and highly communicable from human to human. At present that is not the case, but influenza viruses are notorious for their ability to mutate via a process called antigenic drift.

Mutation into a strain with the potential for pandemic may never happen, but if it does, mortality could be extremely high. Rapid global travel could spread the disease quickly, and, unlike with the seasonal flu strains that come around every winter, our bodies are not immunologically familiar with this type of avian influenza—no one will have native defenses to ward off or minimize infection.

Massive efforts are under way both in the United States and internationally to respond should an avian flu pandemic occur. One of the most important elements in controlling a pandemic will be the development and production of an effective vaccine. Now Yoshihiro Kawaoka and his colleagues at the University of Wisconsin–Madison School of Veterinary Medicine and the University of Tokyo have perfected an advanced method of producing the inactivated “seed” virus used to produce influenza vaccine, a technique known as reverse genetics. This breakthrough may represent a critical step forward in accelerating the production of enough vaccine in a short enough time to prevent massive loss of life.

## The Chicken and the Egg

To be effective, the nonvirulent virus used to make an influenza vaccine must match the genetic makeup of the viral strain that is circulating in the human population. Stimulation of the immune system by exposure to the nonpathogenic form of the viral strain produces antibodies that will confer future resistance to the pathogenic strain. The key is to first identify and then recreate the subtypes of two of the virus’s surface proteins—hemagglutinin (HA) and neuraminidase (NA). These are the “active ingredients” of the virus, determining the strain’s virulence and communicability, and are the targets of vaccine intervention. There are 16 HA subtypes and 9 NA subtypes—the combination of these surface protein subtypes describes the viral strain (for example, H5N1).

Reassortment, the traditional method of seed virus production, has been around for more than 50 years and remains in almost universal use, particularly in the production of annual seasonal flu vaccines. In reassortment, scientists inject fertilized chicken eggs with both a standard nonpathogenic influenza strain known to grow well in eggs and the circulating strain that carries the genes expressing the desired HA and NA protein subtypes. The two viruses multiply, and their genes mix with each other in up to 256 possible combinations of eight genes each. The resultant viruses are then screened, with the desired virus being the one with the six genes that allow the standard strain to grow so well in eggs and the HA and NA genes from the circulating strain. This seed virus is then injected into millions of eggs for mass production of that year’s vaccine.

Edward Janoff, who is chief of the infectious diseases division of the University of Colorado Health Sciences Center School of Medicine and a member of the Infectious Diseases Society of America Pandemic Influenza Task Force, describes the reassortment process as “very tedious.” According to Andrew Pekosz, an assistant professor of molecular microbiology at the Washington University in St. Louis School of Medicine, the whole process to generate seed stocks “could take two weeks optimistically, but more realistically one to two months.” As Kawaoka bluntly puts it, “Classical reassortment? I don’t know why people are still using that method.”

## Monkeying Around with Plasmids

Kawaoka and his colleagues were among the groups who originally developed reverse genetics in the 1990s. With the reverse genetics method, scientists can splice the desired genes—six from the harmless strain and the HA and NA genes from the circulating strain (which have already been adjusted to be nonvirulent)—into small circular pieces of DNA called “plasmids.” The plasmids are then transfected into animal cells, and the vaccine seed virus grows. The seed stock can then be grown in mass quantity for vaccine production either in the traditional chicken egg or in cell culture.

Although the laboratory techniques used in reverse genetics are fairly routine at this point, safety and efficiency issues have presented obstacles to it completely supplanting the reassortment method. The first challenge was the safety of the animal cell line itself. Researchers were concerned that the cells could cause cancer or carry other dangerous viruses. But now a line of African green monkey kidney cells known as Vero cells has been cleared for use in reverse genetics. “These Vero cells have been vetted fairly carefully to be safe,” says Janoff, “and the cell line has now been approved for production of human viruses.”

The second obstacle was the difficulty of transfecting the cell line with plasmids and growing enough virus to be of use as seed stock. “Many of these cell lines that we’d like to use in a cell culture–based vaccine are very hard to transfect with plasmids,” says Karen Lacourciere, an influenza program officer at the National Institute of Allergy and Infectious Diseases. Until now, it was thought to be necessary to transfect eight to twelve plasmids carrying the various viral elements into the Vero cells, and results have been less than ideal in terms of the efficiency of viral rescue—that is, the generation of sufficient numbers of viruses for vaccine use. It can and has been done; the H5N1 vaccine currently in clinical trials (based upon the existing H5N1 strain) was the first one developed via reverse genetics. But clearly, reverse genetics has not been quite ready for prime time.

The refinement to reverse genetics that Kawaoka and colleagues describe in the 15 November 2005 edition of the *Proceedings of the National Academy of Sciences* overcomes this second hurdle. The advance is quite simple. Kawaoka’s group has shown that by combining the viral elements in certain ways, the number of plasmids needed to generate large amounts of virus in Vero cells can be reduced. In short, the team tried several different combinations of genes and numbers of plasmids, until they narrowed down which one seemed to work the best in terms of virus production.

Four plasmids appears to be the ideal number: “If we don’t worry about just generation of virus, we can make a virus with one plasmid,” says Kawaoka. “But in a practical sense, we would use four plasmids, and we would be changing only one plasmid, which encodes HA and NA genes. . . . Our method simply allows one to make vaccine candidate strains easily, so any laboratory can now easily make any H5N1-containing strain.”

## A Small Step with Big Implications

Kawaoka is modest about the achievement, but observers see it as a crucial step forward. “This new reverse genetics system will allow a cell culture–based vaccine strategy to be developed and become more efficient,” says Lacourciere. This is particularly good news given certain problems associated with the egg production system—the need for huge quantities of eggs, and the fact that a significant number of people are allergic to eggs (although no prevalence studies have been done on the general population, 1.5% of children under age 3 are known to have this allergy, according to The Food Allergy & Anaphylaxis Network).

Should a pandemic avian flu strain emerge, time will be of the essence. “What this [method] allows you to do,” says Pekosz, “is generate the seed stock for a pandemic virus twenty-four hours after the pandemic is detected—it could speed up the process that quickly.”

Janoff, who has his finger on the pulse of preparations for a pandemic, agrees. “One of the concerns about a pandemic is that it would spread more quickly than a regular flu,” he says. This means vaccine producers would have a shorter window of time from selecting the virus to having enough vaccine on hand for people both at the source of the epidemic and across the globe as the disease spreads. “So if you can reduce the time from identification and selection to actual vaccine,” he says, “that would really potentially save millions of lives.”

If and when the H5N1 virus mutates into a strain that retains its lethal effects and becomes highly transmissible from human to human, the clock will start ticking, and the race against time to control the pandemic will begin. Thanks to Kawaoka and his colleagues, at least now the human race will have a bit of a head start.

## Figures and Tables

**Figure:**
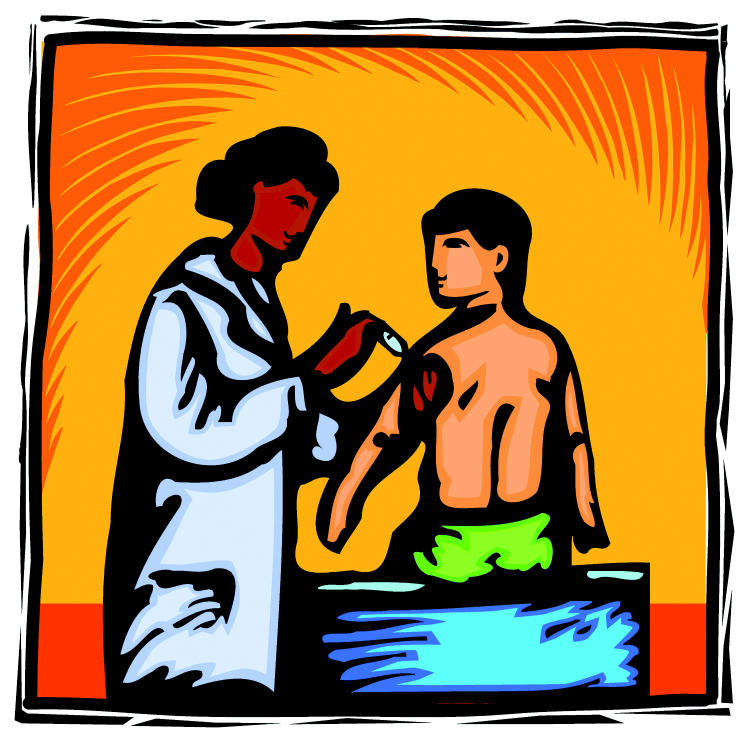


**Figure:**
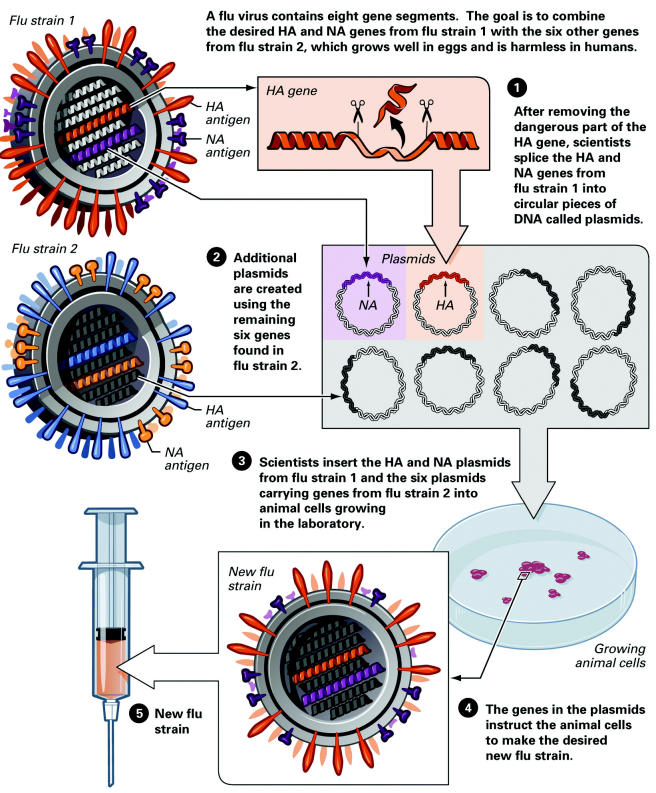
Using Reverse Genetics to Produce Vaccine

**Figure:**
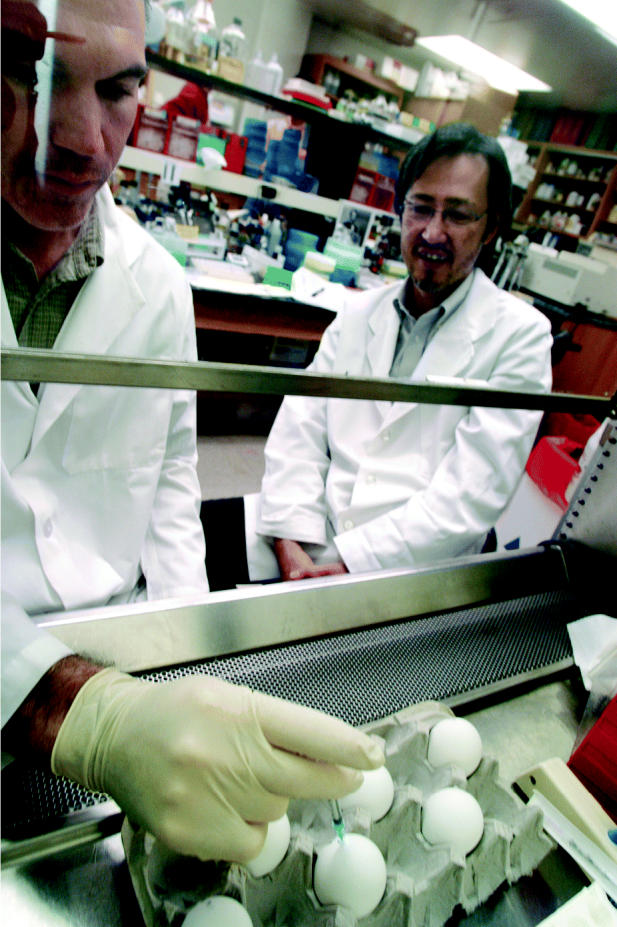
Egg-citing approach to producing vaccines. Yoshihiro Kawaoka (right) and lab technician Barry McClernon (left) oversee an experiment in Kawaoka's laboratory at the UW–Madison School of Veterinary Medicine, where they are working to refine vaccine manufacturing technologies to ensure a faster response to flu outbreaks.
